# Efficacy of submucosal administration of tramadol on acute pain following third molar surgery: a systematic review and meta-analysis

**DOI:** 10.3389/froh.2024.1360298

**Published:** 2024-11-18

**Authors:** Ahmad Salem Assari, Elaf Mubarak Abdullah Algharbi, Abdulmajeed Mohammed Abuhabsha, Basel Basheer Alshammry, Yosef Aeed Alanazi, Reem Abdulaziz Abuhaimed, Ali Mohammad Ali Alzahrani, Abdulrahaman Saud Alduhaim

**Affiliations:** ^1^ Oral Maxillofacial Surgery Diagnostic Sciences Department, College of Medicine and Dentistry, Riyadh Elm University, Riyadh, Saudi Arabia; ^2^ College of Dentistry, King Saud bin Abdulaziz University for Health Sciences, Riyadh, Saudi Arabia; ^3^ College of Dentistry, King Saud University, Riyadh, Saudi Arabia; ^4^ College of Dentistry, University of Hail, Hail, Saudi Arabia; ^5^ Ministry of Health, Riyadh, Saudi Arabia

**Keywords:** analgesic, post-operative pain, opioids, impacted teeth, tramadol, submucosal

## Abstract

**Objectives:**

This systematic review aimed to assess the effectiveness of submucosal tramadol injections in post-operative pain management following third molar surgical extraction.

**Materials and methods:**

Databases, such as PubMed, Scopus, ScienceDirect, and Cochrane Library, were systematically searched using relevant keywords. Randomized clinical trials that met the inclusion criteria were assessed to determine the effectiveness of tramadol in managing acute post-operative pain following third molar surgery.

**Results:**

In total, seven studies with participants of 18 and over following randomized placebo-controlled trials were considered for the analysis. A submucosal injection of 2 ml (50–100 mg) of tramadol adjacent to the impacted mandibular third molar effectively controlled pain for up to 6–24 h following surgery. Non-serious adverse events, such as nausea, vomiting, and headache, were reported in two studies. Meta-analysis (subgroup analysis) revealed heterogeneity among the studies, demonstrating variability in the results across the included studies. In addition, tramadol demonstrated a significant decrease in post-operative pain.

**Conclusion:**

Submucosal tramadol is an efficient, safe, and dependable method for reducing post-operative acute pain, particularly in the first 6 h following impacted third molar surgery. However, due to the observed heterogeneity in the research, there is need for cautious interpretation of the findings and potential limitations in the evidence base. To enhance the quality of evidence on this topic, we strongly recommend conducting new RCTs using established methodologies.

**Clinical relevance:**

Post operative pain following third molar surgeries is one of the common complications. Submucosal tramadol injections were found to be successful in reducing post extraction pain as well as other morbidities.

## Introduction

Post-operative pain is a major complication of third molar extractions, which are one of the most frequently performed dental procedures (1). Third molar extraction is increasingly prevalent in modern dentistry, which leads to pronounced post-operative pain that becomes more severe when the anesthetic agent dissolves. Various methods and medications for reducing post-operative pain following extraction are available (2). Post-surgical pain following third molar extraction can be managed using a combination of nonsteroidal anti-inflammatory drugs (*N*SAIDs), acetaminophen, and local anesthetics. Additionally, multimodal analgesia strategies, such as incorporating corticosteroids and patient education on post-operative care, are employed to optimize pain relief while minimizing opioid usage (3, 4). Some of the pain management methods are related to alternative and complementary medicine (5–8). Third molar surgery is a routine procedure performed by oral and maxillofacial surgeons. It offers benefits including pain relief, caries and periodontal disease prevention, facilitation of orthodontic treatment and orthognathic surgery, and protection against pathological conditions such as dentigerous cyst formation and external root resorption of the adjacent second molar (9).

Mandibular third molar extraction is one of the most commonly performed dental procedures. Since the majority of mandibular third molars tend to be partially impacted, their extraction can be challenging, leading to subsequent post-operative sequelae, such as pain, swelling, and trismus (10). Moreover, transalveolar extraction of impacted mandibular third molars is the most commonly performed oral and maxillofacial surgical procedure with varying post-extraction outcomes. A high incidence of impacted third molars has been reported in Riyadh, Saudi Arabia (11). The reported prevalence of impacted 3rd molar worldwide is around 24.4% (12).

Maxillofacial surgeons routinely perform impacted third molar extraction in private and hospital settings. Moreover, most individuals may need to undergo this procedure at some point. The extraction involves manipulation of the surrounding tissue with respect to the position of the impacted tooth. The extent of bone removal directly affects patient morbidity, with more bone removal resulting in greater discomfort. Moreover, surgical complications, such as swelling, trismus, and discomfort, could impact patients’ daily activities (13–15). Impacted third molars are usually asymptomatic and incidentally found on imaging. The recommendation for the removal of impacted third molars to prevent complications or disease-related issues is a subject of debate. Proponents argue that extraction can prevent potential problems such as impaction-related pain, infection, and damage to adjacent teeth. Once detected, their removal is recommended to prevent further complications or the development of disease-related issues (16, 17). Opponents, however, contend that not all impacted third molars lead to issues and advocate for a more conservative approach, considering the potential risks and benefits of surgery on a case-by-case basis (17, 18).

Post-operative complications of third molar extraction, including pain, trismus, and swelling, have a high occurrence rate and can intensify over time (19, 20). Pain, infection, nerve damage, bleeding, and dry socket are the most commonly reported post-operative complications following third molar extraction; while surrounding tissue damage and trismus occur less frequently (21). Postoperative Symptoms Severity Scale (PoSSe Scale) is frequently used to assess the severity of these sequelae after third molar surgeries. Recent studies have reported this as a valid and reliable method for evaluation (1, 7).

Alveolar osteitis is a condition where the normal healing after extraction does occur as expected. Furthermore, in some cases, the early clot formed in the socket may undergo premature clot necrosis or loss, leading to pain and fetor oris. In addition, alveolar osteitis, dry socket, alveolitis sicca dolorosa, localized alveolar osteitis, and fibrinolytic alveolitis are disturbances that may hinder the healing process occurring after the formation of a mature blood clot and before the replacement of the blood clot with granulation tissue (2).

Transalveolar extraction of third molars is mostly performed traumatically and can result in swelling, limited mouth opening, and moderate-to-severe pain. Severe complications, such as infections and long-term nerve injuries, have a low rate of occurrence. Third molar extractions are accompanied by sequelae of postoperative complications, such as trismus, pain, bleeding, and edema. All these complications are influenced by factors, such as surgical technique, surgeo's experience and skill, and severity of the impaction (2, 20).

The major complications associated with third molar extractions include nerve damage, alveolar osteitis, bacterial infection, bleeding, and pain. Less severe complications include difficulty in mouth opening, iatrogenic damage to the second molar, and iatrogenic fracture of the mandible (22). Effective pain management is the primary goal in oral surgery, especially in anxious patients. A plethora of methods have been used to decrease post-operative pain following third molar extractions. However, submucosal tramadol has demonstrated significant results in managing pain and swelling, as documented by many studies (22).

Pain relief, reduction of possible complications, and healing in a controlled manner should be ensured following the third molar extraction. Analgesics, especially those with an anti-inflammatory action, aid in reducing pain. Dental post-operative pain can be adequately controlled using a variety of non-steroidal anti-inflammatory drugs (NSAIDs). Pain management can play a major role in the recovery of post-operative oral function. Nonetheless, NSAIDs may be contraindicated in patients with peptic ulcers, bleeding disorders, those undergoing anticoagulant therapy, and those allergic to one or more of the ingredients of NSAIDs. Thus, tramadol can be used safely in such patients; moreover, it does not have adverse effects on the respiratory function and consciousness of patients (2).

Pain is the main symptom following third molar removal, and effective pain management is crucial for improving function and quality of life. Pain can be effectively managed using various analgesics, such as NSAIDs; however, NSAIDs are contraindicated in many patients, including those with a history of allergies or peptic ulcers, bleeding diseases, and anticoagulant or steroid use. Tramadol, which is an opioid agonist, has demonstrated effective management of moderate-to-severe pain in both inpatients and outpatients. Effective pain control following oral surgery results in less morbidity and better recovery.

Surgical pain can manifest in several ways, and one novel approach for pain management involves delivering a local anesthetic agent directly to the socket of the tooth instead of using a block or any analgesic agent (NSAIDs). This method employs consistent irrigation with the anesthetic agents. However, patients may experience a needle-prick sensation during the injection of the anesthetic. Moreover, it is associated with a risk of intravascular or intraneural injection of the anesthetic solution, making it essential to take precautions to control and prevent this risk (23). This systematic review and meta-analysis aimed to evaluate the efficacy of submucosal administration of tramadol on acute pain following third molar surgery. The specific aims of this systematic review and meta-analysis are:

1. To evaluate and synthesize existing evidence on the effectiveness of submucosal administration of tramadol injection in managing acute pain following third molar surgery.
2. To assess the overall impact of submucosal tramadol on post-operative recovery, considering factors such as pain intensity, duration, and the occurrence of adverse effects.
3. To provide a comprehensive analysis of the existing literature, identifying trends, variations, and potential biases in studies evaluating the efficacy of submucosal tramadol for pain management after third molar surgery.
4. To offer insights into the practical implications of submucosal tramadol administration, considering its potential benefits and limitations in comparison to other pain management strategies.
5. To contribute evidence-based recommendations for clinicians and researchers regarding the use of submucosal tramadol as a viable option for acute pain control in the context of third-molar surgery.

## Methodology

This systematic review and meta-analysis assess the efficacy of submucosal administration of tramadol injection in managing acute pain following third molar surgery. The study aims to provide a comprehensive evaluation of existing literature, adhering to Preferred Reporting Items for Systematic Reviews and Meta-Analysis (PRISMA) guidelines, to contribute insights into the effectiveness of this pain management approach in the context of post-operative recovery after third molar surgery (24).

### Inclusion criteria

Studies involving patients of any sex, aged >15 years, undergoing third molar extraction; those employing submucosal tramadol injections; and comparative studies (with and without injection) were included for the present meta-analysis. The primary outcome was the reduction in post-extraction pain. The secondary outcome was the reduction in trismus, swelling, and other complications. Additionally, studies conducted in dental clinics or hospitals, as well as randomized controlled trials (RCTs) or observational studies comparing submucosal injection of tramadol with other methods were included.

### Exclusion criteria

Studies comprising patients <15 years or pregnant women; those with no comparative data; those with incomplete data or unclear description of the outcomes; those without controls; case reports, conference reports, animal studies, reviews, theses, and letters; and studies published in languages other than English were excluded. The search was conducted in October 2022.

### Search strategy

Relevant information was collected from numerous databases, including PubMed, Web of Sciences, Scopus, ScienceDirect, Cochrane Library, and Google Scholar, using various keywords and MeSH (Medical Subject Headings) terms. Additionally, search terms such as “third molars,” “wisdom teeth,” “tramadol,” “pain,” and “morbidity” paired with Boolean operators such as “AND,” “NOT,” and “OR” were employed. The detailed keyword search strategy has been described below:

1. Google Scholar: allintitle: “molar*” AND Tramadol
2. Web of Sciences: (“Third molar” OR “Third molars” OR “3rd molars” OR “Wisdom tooth” OR “Wisdom teeth” OR “Third molar surgery”) AND (“Tramadol”) AND (“Pain” OR “Pain management” OR “Acute Pain” OR “Post extraction pain” OR “Trismus” OR “Edema” OR “Swelling” OR “Complications”)

Added suggested Keywords: should include “Dental Pain”, “third molar extraction” and “third molar surgery”. Must include “tramadol”

1. Cochrane Library Trials: all text (“Third molar” OR “Third molars” OR “3rd molars” OR “Wisdom tooth” OR “Wisdom teeth” OR “Third molar surgery”) AND (“Tramadol”) AND (“Pain” OR “Pain management” OR “Acute Pain” OR “Post extraction pain”); Plus all text (“Third molar”) AND (“Tramadol”) AND (“Trismus” OR “Edema” OR “Swelling”)
2. Scopus: (1) (“Third molar” OR “Third molars” OR “3rd molars” OR “Wisdom tooth” OR “Wisdom teeth” OR “Third molar surgery”) AND (“Tramadol”) AND (“Pain” OR “Pain management” OR “Acute Pain” OR “Post extraction pain”); Plus (2) (“Third molar” OR “Third molars” OR “3rd molars” OR “Wisdom tooth” OR “Wisdom teeth” OR “Third molar surgery”) AND (“Tramadol”) AND (“Trismus” OR “Edema” OR “Swelling” OR “Complications”) filter: RCT only and Articles only, within Article title, abstract and keywords
3. PubMed: (“Third molar*” OR “Wisdom teeth*” OR “3rd molar*” OR Molar* OR “tooth*”) AND (extraction* OR Surgery*) AND (“Tramadol”) AND (“Pain” OR “Trismus” OR “Swelling*” OR “Complication*” OR “infection*” OR “adverse effect*”)
4. ScienceDirect: (“Third molar” OR “3rd molar” OR “tooth”) AND (extraction OR Surgery) AND (“Tramadol”) AND (“Pain” OR “Trismus” OR “Swelling”)

Filters used: Research articles, Encyclopedia, Practice guidelines and English only.

### Assessment

The primary articles were reviewed and the titles and abstracts of the studies were screened independently. Two researchers reviewed the articles during the initial search and later evaluated the full-text articles. Both researchers separately reviewed the methodology and were unaware of each othe's decisions. Any disagreement between the two researchers was discussed and resolved by consensus. If a conflict could not be resolved, a third researcher was consulted. The “Risk of Bias” table within RevMan 5.4 was used to systematically assess the risk of bias in individual studies included in this systematic review and meta-analysis. This table allowed reviewers to document their assessment of various domains of bias for each included study. The domains assessed included random sequence generation, allocation concealment, blinding of self-reported outcomes, blinding of objective outcomes, blinding of participants and personnel, blinding for outcome assessment for self-reported outcomes or objective outcomes, incomplete outcome data, selective reporting, and other biases.In the “Risk of Bias” table, each domain was evaluated and categorized as “low risk,” “high risk,” or “unclear risk” of bias, based on the information provided in the study report. Reviewers consider factors such as study design, methodology, and conduct to make judgments about the risk of bias in each domain.

### Data extraction

The selected studies that satisfied the inclusion criteria underwent a quality assessment prior to data extraction. Screening was performed for the titles, abstracts, and full texts of the papers, and the extracted data were entered into a standardized data extraction form. The following information was extracted: study characteristics, including authors, year of study, affiliation, country of origin, and study design; participants’ characteristics, including sample size, age, sex, and other useful information; intervention; outcome measures; results; adverse events; materials and methods; tooth condition, evaluation method; follow-up; and conclusion. Reasons for exclusion were recorded and reported in the Preferred Reporting Items for Systematic Reviews and Meta-Analysis (PRISMA) flowchart.

### Data analysis

The articles included in the systematic review were summarized using qualitative analysis. For any outcomes that were consistent across the selected studies, RevMan 5.4 (25) was used to determine the Cochrane Q and *I^2^* values, which measured the dispersion between trials. The significance threshold was set at 0.05, and a random-effects model was employed. For selected investigations, a critical evaluation of the results was also considered. Finally, the RCTs were evaluated to measure bias risk using the Cochrane technique (ROB 2.0) (26).

### Heterogeneity assessment

Heterogeneity was evaluated using the *I^2^* test. Interpretation of *I^2^*test was performed according to the Cochrane Handbook for Systematic Reviews of Interventions (25), as follows:

A) 0%–40%: indicates minimal or possibly not significant heterogeneity among the studies
B) 30%–60%: could indicate moderate heterogeneity
C) 50%–90%: may indicate significant heterogeneity
D) 75%–100%: significant heterogeneity

The significance of the observed I*^2^* value may be influenced by the quantity, direction, and strength of evidence for heterogeneity (e.g., *P*-value from the chi-square test or a confidence interval for *I^2^*).

Additionally, a concise summary of the main outcomes of interest, along with the quality of evidence for each outcome was presented as a Summary of Findings (SoF) in a Table and a narrative description of the quality of evidence for each outcome was provided.

## Results

**Figure 1:**
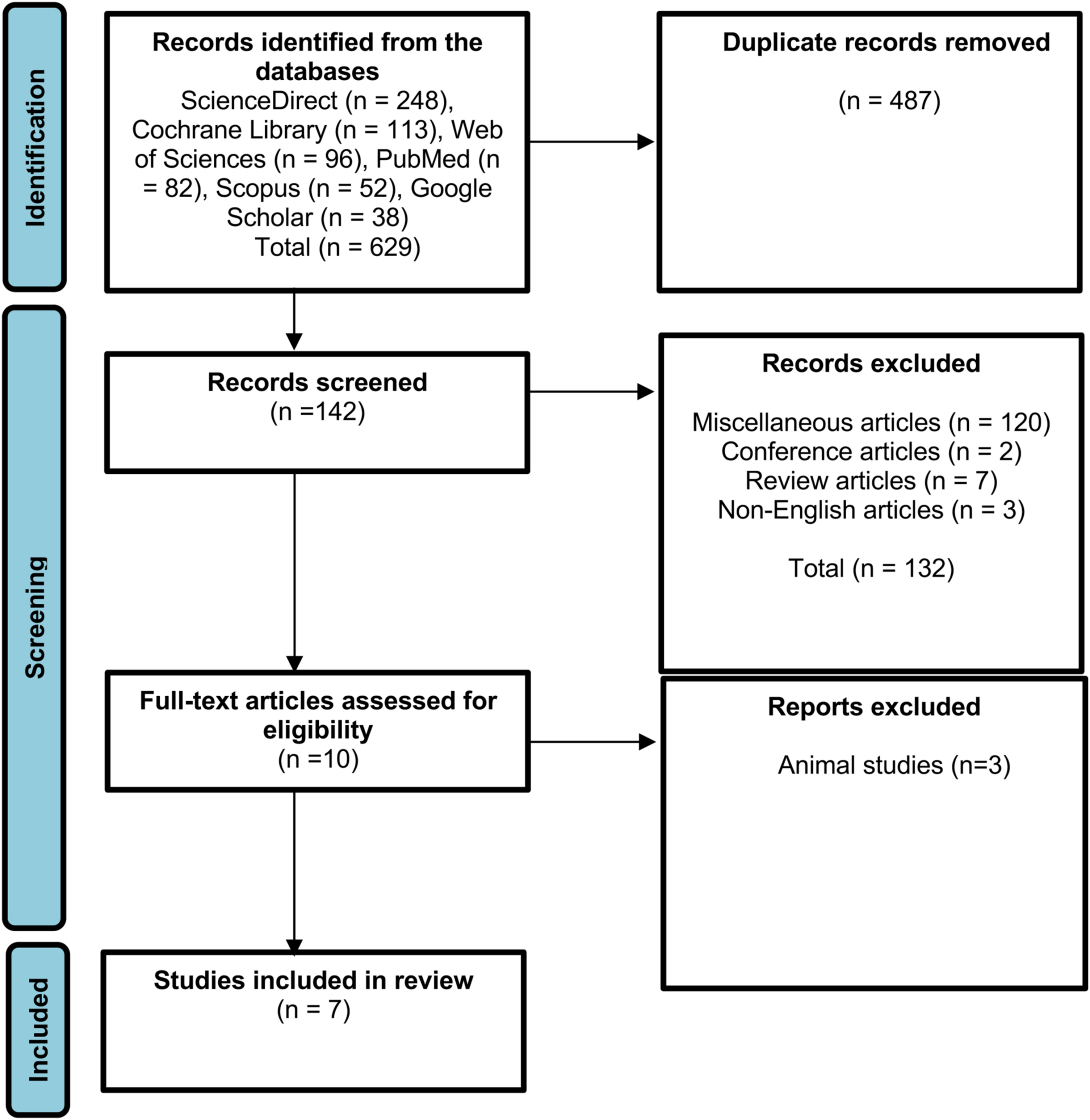
Preferred reporting items for systematic reviews and meta-analysis (PRISMA) flowchart.

To identify relevant literature, several data sources were examined, and 629 articles were identified using Scopus, ScienceDirect, PubMed, Cochrane Library, Web of Sciences, and Google Scholar. In total, 487 duplicate articles were eliminated after initial screening, and the remaining 142 articles underwent additional screening according to the inclusion criteria. Additionally, 132 articles were excluded because they did not meet the inclusion criteria. Out of the 10 eligible studies selected for full-text assessment, 3 of them employed animals as test subjects. Finally, seven studies were selected for further investigation (Figure 1).

### General characteristics of the included studies

Most of the included studies were reported from India (19, 27, 28), whereas two studies from Pakistan (29, 30) and one study each from Turkey (31) and Brazil were reported (32). Six studies employed a blinded RCT study design (19, 28–32), whereas one study used a prospective study design (27) with the aim of evaluating the efficacy of submucosal application of tramadol injection following third molar extraction. The participants in the included studies had a mean age of 22–30 (range: 8–84) years. No dropouts were reported in any of the studies; five studies included both male and female participants (19, 28–30, 32), whereas two studies did not mention the sex of the participants (27, 31). Two studies reported that patients with an ASA I–III classification visited the clinic for third molar extraction (28, 31), whereas five studies reported normal third molar extraction (Table 1) (19, 27, 29, 30, 32).

**Table 1:** General characteristics of the included studies.

Bibliometric properties		Study design						Participants		
Author (year)	Country	Aim	Study setting	Recruitment period	Number of participants	Groups (*N*)	Dropouts	Age	Sex	Other relevant information
Gonul et al., (2015) (31)	Turkey	Evaluation of the effectiveness of tramadol, for acute post-operative facial pain following the extraction of impacted third molar.	Prospective, double-blind, RCT	NA	*N* = 60	Intervention = 30 Control = 30	0	Intervention: 24.80 ± 2.524 Control: 23.93 ± 2.828	NA	ASA I–II patients undergoing impacted third molar surgery
Ceccheti et al., (2014) (32)	Brazil	To assess the analgesic and adjuvant anesthetic effects of submucosal tramadol after third molar extraction.	Prospective, double-blind, split-mouth, placebo-controlled, single-dose, crossover	NA	*N* = 52	Not mentioned	Not mentioned	22.10 ± 3.84 (18–33)	Male = 16 Female = 36	NA
Feroz et al., (2022) (29)	Pakistan	To determine the effectiveness of submucosal application of tramadol.	Randomized controlled trial	January 1, 2021–December 31, 2021	*N* = 84	Group A = 42 Group B = 42	0	Group A: 25.7 ± 4.7 (18–35) Group B: 26.5 ± 4.9 (18–35)	Group A: Male = 25 (59.5%) Female = 17 (40.5%) Group B: Male = 25 (59.5%) Female = 17 (40.5%)	Patients requiring impacted mandibular third molar extractions
Ghafoor et al., (2016) (30)	Pakistan	To determine the analgesic effects of sub mucosal tramadol following third molar extraction.	Randomized, double blind placebo control clinical trial	February 23, 2015–June 30, 2015	*N* = 60	Group A = 30 Group B = 30	0	30.17 + 2.532 years (25–35 years)	Male = 39 Female = 21	Patients who underwent surgical extraction of one of their impacted mandibular third molars
Panchal et al., (2019) (27)	India	To determine the effect of submucosal injection of tramadol after surgical extraction of third molar and its implication over swelling and mouth opening.	Prospective study design	NA	*N* = 8	Group A = 8	0	NA	NA	NA
Iqbal & shetty (2019) (19)	India	To evaluate the effectiveness of submucosal injection of tramadol in treating post-operative pain after surgical extraction of impacted mandibular third molars.	Randomized controlled trial	November 2016–September 2018	*N* = 60	Intervention = 30 Control = 30	0	27.78 years (19–45 years)	Male = 32 Female = 28 Intervention; Men = 15 Women = 15 Control; Men = 17 Women = 13	Patients reporting to the clinic for surgical extraction of impacted mandibular third molars
Mazhar et al., (2020) (28)	India	To assess preemptive analgesic efficacy of oral ketorolac with submucosal placebo vs. oral ketorolac with submucosal tramadol during impacted mandibular third molar surgery.	Double-blind, comparative clinical trial	January 2015–October 2016	*N* = 40	Group A = 40 Group B = 40	0	22.10 ± 3.15	Male = 24 Female = 16	Patients with ASA grade I category, having asymptomatic bilateral identical impacted mandibular third molars along with grade II or III difficulty of extraction

NA, not available; RCT, randomized controlled trial; ASA I and II, American Society of Anesthesiologists classification I and II.

### Intervention and outcomes

The summary of intervention and outcomes is presented in Table 2. In all the studies, tramadol injections of different doses were submucosally administered (19, 27–32); one study also used 10 mg ketorolac orally along with tramadol injection (28). Normal saline was used as the control. The primary outcome measured in all the studies was post-operative pain, whereas secondary outcomes included the first analgesic dose time, total dose, and adverse tissue reaction (19, 28, 30, 31). Time of ingestion, amount of analgesic rescue medication, and length of anesthetic blockade were reported in one study (32), whereas swelling and trimus were reported in two studies (27, 29). The visual analog scale (VAS) was employed in six studies to measure the level of pain following tramadol injection following extraction, whereas one study used the Wong–Baker FACES Pain Rating Scale (27, 29); measurements in all the studies were performed using a self-reported checklist following the time set by the researchers. The studies used different time points to measure the VAS scores, as shown in Table 2. There was a limited to two types of scales to assess Patient-Reported Outcomes (PROs). Assessing patient-reported outcomes (PROs) is crucial for evaluating the influence of interventions on patients’ quality of life and holistic well-being (33). The outcome measure tool for assessing oral health-related quality of life (OHRQoL) is a crucial component in evaluating the impact of oral health interventions on individuals’ overall well-being and satisfaction with their oral health status (33).However, none of the studies used any oral health-related Quality of Life (OHRQoL) Outcome measure tool.

**Table 2:** Interventions and outcomes of the included studies.

Bibliometric properties	Intervention		Outcomes & measures				
Author (Year)	Intervention	Groups	Primary outcome	Measures	Time points	Secondary outcomes	Measures
Gonul et al., (2015) (31)	1 mg/kg tramadol injection	2-ml saline	Post-operative pain	VAS	1, 2, 4, 6, 24, and 28 h	Time of intake of first analgesic, total analgesic dose, adverse tissue reaction	Self-reported measure
Ceccheti et al., (2014) (32)	2 ml of 100 mg tramadol injection	Normal saline solution	Post-operative pain intensity	VAS	4, 8, 24, 48, and 72 h.	Anesthetic blockade duration, time of intake, amount of analgesic rescue drug	Self-reported measure of the amount of analgesic consumption and time elapsed between the onset of the anesthetic effect and restoration of normal lip sensation to determine the duration of the sensory blockade
Feroz et al., (2022) (29)	100 mg/2 ml tramadol injection	Sterile 2 ml of 0.9% normal saline	Post-operative pain	Wong–Baker FACES Pain Rating Scale	Day 1, 2, 3, and 7	Swelling, trismus	Swelling: angle of the mandible to the menton (ranging from 2 mm to 8 mm) Trismus: Mild/grade I (35–26 mm), Moderate/grade II (25–16 mm), Severe/grade III (15–0 mm)
Ghafoor et al., (2016) (30)	100 mg/2 ml tramadol injection	Normal saline solution	Post-operative pain	VAS	4, 8, 24, and 28 h.	Time of intake and amount of analgesic rescue drug	Self-reported measure
Panchal et al., (2019) (27)	50 mg tramadol injection	NA	Post-operative pain	VAS	4, 8, and 24 h.	Swelling and mouth opening	Self-reported measures
Iqbal & shetty (2019) (19)	50 mg tramadol injection of 1 ml solution	1-ml saline solution injection	Post-operative Pain	VAS	0.5, 1, 2, 4, 6, 12, 24, and 48 h.	Time of intake of the first analgesic	Self-reported measure
Mazhar et al., (2020) (28)	10 mg oral ketorolac with 50 mg submucosal local tramadol (1-ml solution)	10 mg oral ketorolac with submucosal local placebo (1 ml saline solution)	Post-operative pain	VAS and verbal response scale	1, 2, 3, 4, 6, and 12 h.	Time until the 1st rescue analgesia intake, need of analgesic intake during the first 24 h postoperatively, and patien's experience	Self-reported measure

NA, not available; VAS, visual analog scale.

## Results and adverse events

Based on the results of the included studies, the overall VAS scores were observed to be higher in the control group than in the tramadol group, especially during the first 24 h (*P* < 0.05) (19, 27–32); an insignificant (*P* > 0.05) difference was observed in the VAS scores after 24 h (31). Trismus-related statistical differences were only observed on day 1, whereas swelling demonstrated statistically significant values at 24 and 72 h following extraction. Additionally, statistically significant mouth opening was observed at every hour of the day (27, 29) as shown in Table 3. Five studies did not report any adverse events occurring during the treatment (19, 27, 29, 30, 32), whereas two studies reported adverse events including nausea, vomiting, and headache (Table 3) (28, 31). Before third molar extraction, the condition of the third molars were examined. Three of the studies reported completely impacted molar (27, 31, 32) and other three studies reported impacted molar teeth, classified as Pell and Gregory class 2 and 3, position B and Winters mesioangular position (28–30), whereas one study did not report the condition of the molars (19). Overall, all the studies demonstrated the effectiveness of tramadol in controlling post-operative pain, as shown in Table 3.

**Table 3:** Results and adverse events reported in the included studies.

Bibliometric properties	Results			Conclusion
Author (Year)	Results	Adverse events	Teeth condition	
Gonul et al., (2015) (31)	The VAS scores of the control group (group S) 1, 2, 4, 6, and 12 h postoperatively were significantly higher than that of the tramadol group (group T). No significant group differences were observed in the VAS scores 24 and 48 h postoperatively (*p* > 0.05). First analgesic was administered significantly later in the tramadol group than in the control group (*P* = 0.0001). Total analgesic intake in the control group was significantly higher (*P* = 0.0001).	No significant group differences were observed in terms of side effects (nausea, vomiting, burning, and dizziness)	Third molar was completely impacted and moderately angulated	Submucosal tramadol injection is an efficient, safe, and dependable method to reduce acute post-operative facial pain. Additional research is warranted to confirm the effectiveness of submucosal tramadol following dental or surgical operations.
Ceccheti et al., (2014) (32)	Patients in the tramadol group less frequently used the rescue analgesic than those in the placebo group (*P* = 0.008) (control: 4.4 ± 93.71; intervention 3.37 ± 4.65). The mean time until the first analgesic requirement in the tramadol group was significantly longer than that in the placebo group (*P* = 0.006) (control: 185.4 ± 59.4, treatment: 303.72 ± 416.01). The VAS score was observed to differ significantly between the two groups only when patients felt that anesthesia had worn off (*P* = 0.001). Later evaluations revealed no differences in the mean pain values between both groups.	No	Mandibular-impacted third molars	Submucosal tramadol injection improves postoperative analgesia but does not lengthen the duration of the anesthetic activity after oral surgery.
Feroz et al., (2022) (29)	A statistically significant difference was observed in pain and swelling between patients in groups A and B on day 1, 2, and 3; however, no significant results were observed on day 7. Statistical difference in terms of trismus was observed only on day 1.	No	Impacted mandibular third molar extractions. Pell and Gregory class 2, position B. Winters mesioangular position.	Submucosal tramadol is superior to sterile normal saline in terms of its effectiveness, safety, and dependability in patients who have undergone third-molar surgery. When compared to sterile normal saline, tramadol has a much lower rate of post-operative discomfort, edema, and trismus. The severity of trismus is also minimal.
Ghafoor et al., (2016) (30)	The patients in the intervention group had less pain intensity (M = 4.73) than that of the patients in the control group (M = 6.0). Moreover, patients in the treatment group had longer time elapsed before taking rescue medication and took lesser number of pills than those of the patients in the control group.	No	Third molar extractions on the mandible, Pell and Gregory class II position B. Winters mesioangular position.	As compared to other oral analgesics, such as NSAIDs, the results of the current study suggest that local administration of 100 mg/2 ml tramadol provides a prolonged pain-free period (approximately 5–6 h) with rare adverse effects and is a safe medication to be used for post-operative analgesia following dentoalveolar surgery.
Panchal et al., (2019) (27)	A statistically significant VAS score for pain was noted following submucosal tramadol injection in surgical extraction of the mandibular third molar at 4, 8, and 24 h. Statistically significant values were observed for swelling at 24 and 72 h following extraction. Additionally, statistically significant values were observed at 24 h for mouth opening.	No	The third molar location was categorized after being assessed in a panoramic radiograph.	Submucosal tramadol following mandibular third molar extraction successfully reduces pain, post-extraction swelling, and improves on mouth opening by inducing fewer problems and avoiding stomach disturbances.
Iqbal & shetty (2019) (19)	Post-operative pain scores, as recorded on the VAPS, were significantly lower in the case group (group A), receiving tramadol injection, than in the control group (group B) and the differences were statistically significant at 0.5, 1, 2, 4, and 6 h ([M = 0.933, SD = 0.944; *P* = 0.001], [M = 2.100, SD = 1.539; *P* = 0.006], [M = 2.200, SD = 1.955, *P* = 0.001], [M = 2.400, SD = 1.850; *P* = 0.001], and [M = 2.200, SD = 1.126; *P* = 0.001, respectively). The mean time at which the first tablet was taken was 3.23 h in the tramadol group and 1.97 h in the control group (*P* = 0.001). The mean total number of tablets received by groups A and B were 6.67 and 8.23, respectively (*P* = 0.001).	No	NA	Surgical removal of impacted third molars considerably impacts post-operative pain control when tramadol is administered submucosally.
Mazhar et al., (2020) (28)	The patients experienced significantly lower pain intensity scores from the 1st to 12th post-operative h with oral ketorolac plus local tramadol in group A as compared to group B who received oral ketorolac plus local saline as placebo. According to the VAS, patients in group A experienced significantly lower pain in 3, 4, and 6 h than those in group B Mean time to take rescue analgesia: the pain-free interval was observed to be significantly longer in group A (6.96 ± 1.47 h) (highly significant; *P* = 0.001) than in group B (4.59 ± 0.99 h) Total analgesic consumed: highly significant difference (*P* = 0.001) was observed with mean value at 1.29 ± 0.45 and 2.53 ± 0.66 for groups A and B, respectively.	Headache, nausea, and vomiting	Impacted mandibular third molars with extraction difficulty grades II or III	For the management of acute pain following surgical removal of third molars, preemptive use of oral ketorolac combined with topical tramadol is more tolerated than ketorolac. With oral ketorolac and local tramadol, the patients had significantly decreased pain scores in the early post-operative period.

VAS, visual analog scale; NSAIDs, non-steroidal anti-inflammatory drugs; M, mean; SD, standard deviation.

### Meta-analysis

**Figure 2:**
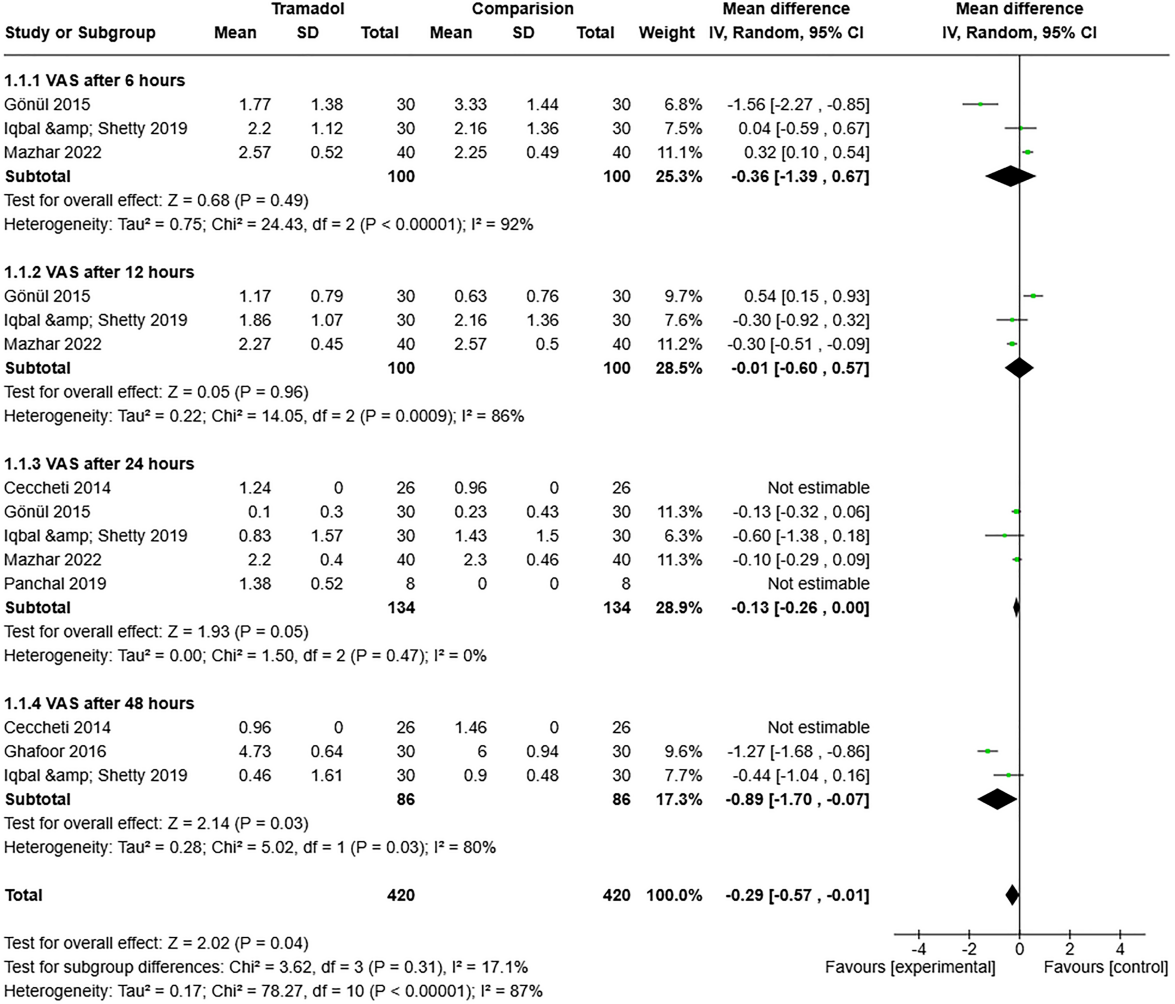
Forest plot of the pain intensity (after 6, 12, 24, and 48 h) in the tramadol injection and comparison (control or placebo) groups after impacted third molars surgery.

The VAS scores after 6, 12, 24, and 48 h were meta-analyzed to assess heterogeneity among the studies. After 6 h, the combined findings for pain intensity from the seven studies revealed a high degree of variability among them (*I^2^* = 92%, *P* = 0.00001). The outcomes were combined using a random-effects model. As seen in Figure 2, a significant difference and correlation was observed between the tramadol injection and comparison groups (control or placebo) (RE 95% confidence interval [CI] = −0.36 [−1.39, 0.67], *P* = 0.00001). The pooled results for pain intensity after 12 h from the seven studies demonstrated significant inter-observer variation (*I^2^* = 86%, *P* = 0.0009). The results were amalgamated using a random-effects model. The tramadol injection administration group differed significantly from the comparison groups (control or placebo) (RE [95% CI] = −0.01 [−0.60, 0.57], *P* = 0.0009), as shown in Figure 2. However, the diversity in the pooled results for pain intensity after 24 h from the seven studies was minimal (*I^2^* = 0%, *P* = 0.49). The results were amalgamated using a random-effects model. The tramadol injection and comparison groups (control or placebo) did not differ significantly (RE [95% CI] = −0.13 [−0.26, 0.00], *P* = 0.49), as shown in Figure 2. The variability between them was still high (*I^2^* = 80%, *P* = 0.03) in the pooled data for pain intensity after 48 h from the seven studies. A random-effects model was used to aggregate the results. Figure 2 shows a significant difference and association between the tramadol injection and control or placebo groups (RE [95% CI] = −0.89 [−1.70, −0.07], *P* = 0.03).

Overall, heterogeneity was observed among the included studies (*I^2^* = 87% and *P* = 0.00001). However, the test for subgroup differences was non-significant (*I^2^* = 17.1% and *P* = 0.31) (Figure 2).

### Risk of bias

**Figure 3:**
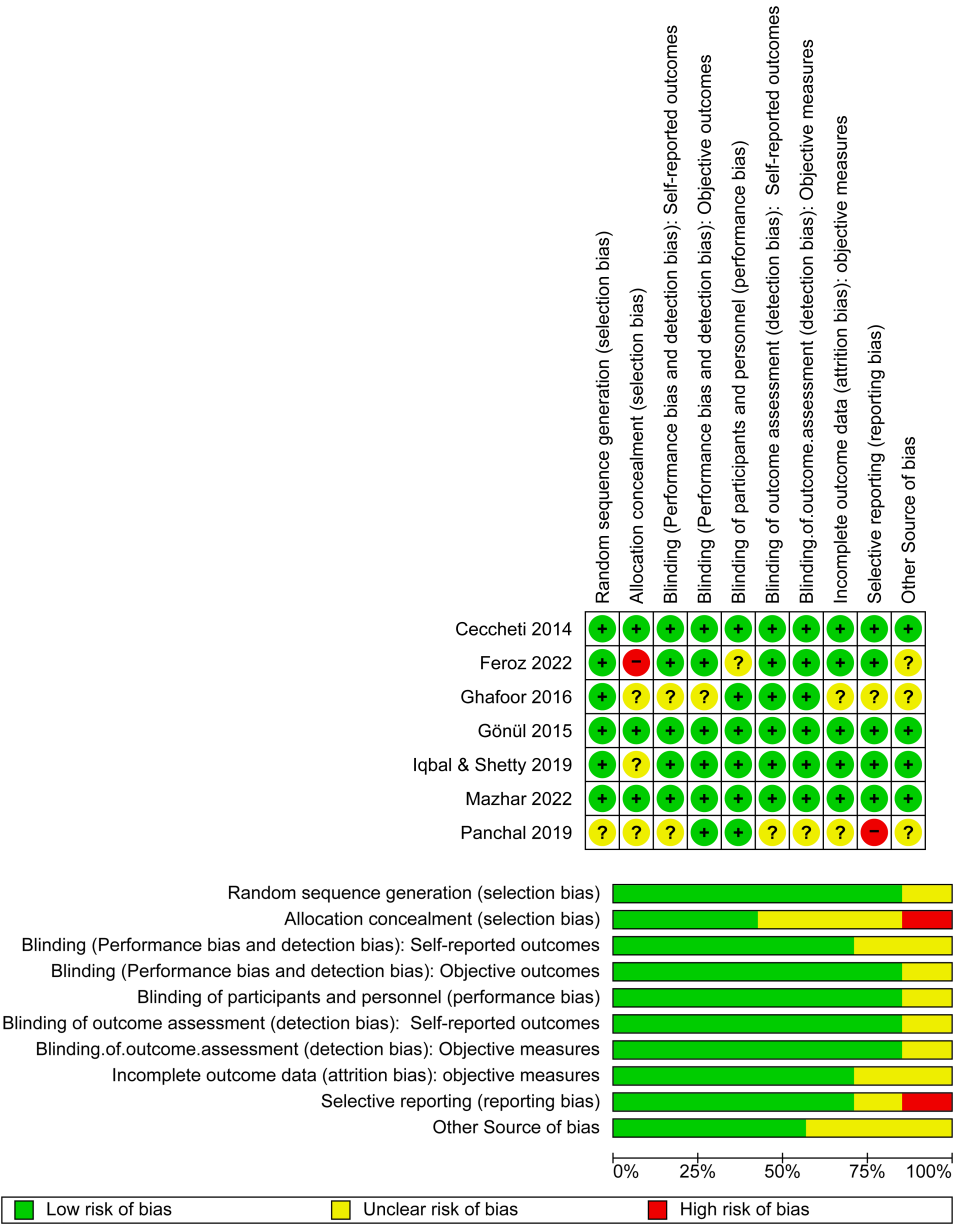
Risk of bias graph and summary of the included studies.

In RevMan 5.4, the “Risk of Bias” table was used to assess the quality of the included studies. A judgment (“low risk,” “high risk,” or “unclear risk” of bias) was applied to each input. Most of the studies exhibited a low risk of bias in terms of randomization, except for one study (27), which exhibited an unclear risk. In the allocation concealment, low, unclear, and high risk of bias was reported in three (28, 31, 32), three (19, 27, 30) and one study(s), respectively (29). In the blinding assessment, five studies exhibited a low risk (19, 28, 29, 31, 32) and two studies exhibited an unclear risk (27, 30); other domain assessments are shown in Figure 3. Overall, all the studies demonstrated good quality.

## Discussion

The findings of this systematic review and meta-analysis provide valuable insights into the efficacy of submucosal tramadol administration for managing acute post-operative pain following third molar surgery. When interpreting these results, it is essential to consider them in the context of existing evidence and the broader landscape of pain management strategies in oral surgery. Submucosal tramadol administration has emerged as a promising approach for pain control in the immediate post-operative period, with significant reductions in pain intensity observed, particularly within the first 6 h after surgery. These findings align with previous research demonstrating the analgesic efficacy of tramadol in various surgical settings. However, it is essential to note that the heterogeneity observed among the included studies underscores the need for cautious interpretation of these results. Nonetheless, the overall trend toward improved pain management with submucosal tramadol is encouraging and warrants further investigation.

Systematic reviews and meta-analyses have become increasingly prevalent in the field of medicine (34), making the development of new clinical practice guidelines and future research more convenient, thereby benefitting both clinicians and researchers (35). These approaches allow for identifying, choosing, synthesizing, and evaluating high-quality research data to offer a high-level summary of a specific research or clinical questions (36). The present study aimed to provide an objective summary of the effectiveness of submucosal tramadol administration in managing acute post-operative dental pain following surgical extraction of third molar. Clinical techniques for submucosal tramadol administration include injection, infiltration, nerve block, topical application, and sustained-release formulations (32).Injection involves direct delivery of tramadol solution into submucosal tissue at the surgical site. Infiltration administers tramadol into surrounding submucosal tissue for localized analgesia. Nerve block targets specific nerves to block pain signals, commonly used in dental surgeries. Topical application applies tramadol gel or cream onto mucosal surfaces for transmucosal absorption. Sustained-release formulations offer prolonged analgesic effects postoperatively. Technique selection depends on clinical context, patient needs, and procedure nature. Consideration of appropriate technique is crucial for desired outcomes and patient characteristics. Submucosal tramadol administration provides effective pain management in various clinical scenarios. Tramadol administration for analgesia following surgery has been reviewed in numerous trials; however, few studies have examined its submucosal administration. Only one trial had documented the submucosal tramadol administration following pediatric tonsillectomy surgery and demonstrated a reduced need for post-surgical analgesia (37). The present study focused, exclusively on RCTs that included a control or placebo group for the analysis. RCTs were included because they provide an equal opportunity for selection of both male and female patients for either treatment or control groups, thereby reducing the chances of selection and randomization bias. Moreover, the study strictly adhered to the PRISMA statement (38), which ensured transparency and clarity in the conducted systematic review. Post-operative pain following third molar extraction is a common study model in efficacy trials because of the association of impacted third molars with caries, pericoronitis, periodontal abnormalities in the distal surface of second molars, odontogenic cysts, and dental crowding, necessitating extraction (39). Moreover, third molar surgery is one of the most common dental procedures, which could provide a sufficient number of patients to conduct a research (40). Tramadol, like lidocaine, has a blocking effect that reduces ectopic activities in hypersensitive neurons and suppresses neuronal transmission, making it suitable for reducing pain intensity. Both lidocaine and tramadol demonstrate usage-dependent blockade and have a high affinity for rapidly inactivated sodium ion channels compared to resting channels (41). The present study included both male and female patients of all age experiencing post-operative pain, as the literature has suggested that several variables, such as the complexity of the treatment, patien's age and sex, and the surgeo's experience, can influence post-operative pain (42).

Post-operative pain following the extraction of an impacted third molar is frequently used to assess the analgesic efficacy due to the consistency and intensity of the pain (43). The present study revealed that submucosal injection of 2 ml (50–100 mg) of tramadol adjacent to the impacted mandibular third molar was effective in reducing pain for up to 6 h following surgery, with positive effects continuing for up to 24 h. This finding is consistent with that of a previous systematic review, supporting the effectiveness of this approach. This procedure also aids in starting rescue analgesics sooner and using less rescue analgesics overall (44). In addition, the effects of tramadol administration, both systemically and locally (submucosally), on pain relief following the extraction of an impacted mandibular third molar were examined in another study. The findings revealed that tramadol (50 mg) injected locally into the surgical site greatly improves post-operative analgesia and significantly extends the duration of the anesthetic effect (45). In one study, a combination of tramadol with 10 mg ketorolac demonstrated a positive effect in reducing pain as a combination of analgesics can have a “sparing effect,” enabling pain relief with lower doses and fewer adverse effects. The World Health Organization recommends using a combination of drugs because of the presence of numerous nociception pathways *n* the human body (46). Similar findings were reported by Isiordia et al. and Kim et al. who reported that both medicines have substantial analgesic effects (47, 48). In the present review, only two studies reported adverse events in the form of nausea, vomiting, and headache. Although sleepiness is also a side effect of 75 mg tramadol, unpleasant reactions, including nausea and vomiting, are more common (49). Burning, discomfort, and localized pre-anesthetic erythema may occur even at the injectable dose of 50 mg tramadol (50). Tramadol was similarly or less tolerated by patients following submucosal injection than the control drug, indicating that the drug has a low incidence of side effects and the possibility of a nocebo effect (51). Therapeutic doses of tramadol, even when applied locally, do not impair the respiratory or circulatory systems (52). Tramadol showed lesser analgesic efficacy and a higher incidence of adverse effects compared to NSAIDs in a systematic review that evaluated oral and intramuscular routes of tramadol for third molar pain (53). Moreover, their findings indicated that a single dose of tramadol was not as effective or as safe as NSAIDs for the relief of pain following third molar operations.

Meanwhile, question can arise why injectable tramadol and possible explanation can be that injecting tramadol submucosally allows for a more rapid onset of action compared to oral administration, ensuring quicker pain relief during the critical postoperative period. Submucosal administration bypasses the gastrointestinal tract, avoiding potential delays associated with oral absorption, especially if the patient experiences nausea or has delayed gastric emptying after surgery (20). Moreover, submucosal injection allows for a more precise and controlled delivery of the drug directly to the site of action, maximizing its effectiveness.

A qualitative systematic review by Gounari et al. (54) assessed the impact of both parenteral and submucosal applications of tramadol on perioperative pain management of third molar extraction. The study showed submucosal infiltration of tramadol enhanced analgesic effects, particularly when used in conjunction with oral ketorolac, thus providing a viable alternative to conventional NSAID therapy. These findings underscore tramado's potential as a flexible analgesic that can be effectively tailored to individual patient needs, particularly for those who may not tolerate NSAIDs well (54).

The current meta-analysis revealed that tramadol administered submucosally 6 h after third molar surgery significant improved pain management compared to a placebo. This could be explained by the fact that tramadol has a 6-h plasma half-life, regardless of the method of administration, indicating that this medication has positive effects, particularly in early pain control. In contrast, placebo was more effective than submucosal tramadol in controlling pain 24 h after surgery. This was probably because the effect of tramadol was reduced by half, causing its analgesic effect to decrease over time. Additionally, the local application of the drug resulted in its gradual absorption into the systemic circulation. Another systematic review also reported a reduction in the analgesic effect of tramadol compared to a placebo, 12 h after surgery (44). Although heterogeneity was observed among the studies after 48 h of extraction, a positive effect of tramadol was still evident compared to the control or placebo. This may be attributed to the relatively small number of studies included in this review. Summary measurements of pain from 6 to 24 h after surgery revealed a significant difference in the VAS scores, favoring the tramadol group.

## Limitations

The overall strength of evidence for the use of submucosal tramadol in managing postoperative pain following third molar surgery was rated as moderate. This means that the evidence is considered to be of reasonable quality and is likely to be reliable, but there are some limitations or uncertainties that may affect the confidence in the findings.

The main limitations of the evidence included the relatively small number of studies included in the analysis, the heterogeneity among the studies, and the potential risk of bias in some of the included studies. However, the consistency of the findings across multiple studies and the overall direction of the effect size provided some confidence in the results.

In our opinion, the evidence for clinical decision-making is constrained by the number of clinical trials that have been examined, lack of standardization in the classification system for third molar impaction, level of surgical difficulty, length of the procedure, and surgeo's experience. The relatively small number of research articles with a small sample size may also be a limitation of this study; thus, the findings may not be suitable in the decision-making process.

## Conclusion

Submucosal tramadol is an efficient, safe, and dependable method for reducing post-operative acute pain, particularly in the first 6 h following impacted third molar surgery. Additionally, submucosal tramadol decreased the need for rescue analgesics and combination therapies. Notably, no serious adverse events were reported, and summary VAS pain assessments from 6 to 24 h after surgery revealed a substantial difference in favor of the tramadol group. Based on the moderate strength of evidence, it can be concluded that submucosal tramadol is an effective and safe option for managing postoperative pain following third molar surgery. However, due to the observed heterogeneity in the research, caution must be exercised when interpreting the results of this study. To enhance the quality of evidence on this topic, we strongly recommend conducting new RCTs using established methodologies to address the limitations of the existing evidence, such as the heterogeneity among the studies and the potential risk of bias.

## Data Availability

The original contributions presented in the study are included in the article/Supplementary Material, further inquiries can be directed to the corresponding author.
